# Differential Surface Expression of ADAM10 and ADAM17 on Human T Lymphocytes and Tumor Cells

**DOI:** 10.1371/journal.pone.0076853

**Published:** 2013-10-09

**Authors:** Henriette Ebsen, Alexandra Schröder, Dieter Kabelitz, Ottmar Janssen

**Affiliations:** University of Kiel, Institute for Immunology, University Hospital Schleswig-Holstein Campus Kiel, Kiel, Germany; New York University, United States of America

## Abstract

A disintegrin and metalloproteases (ADAMs) have been implicated in many processes controlling organismic development and integrity. Important substrates of ADAM proteases include growth factors, cytokines and their receptors and adhesion proteins. The inducible but irreversible cleavage of their substrates alters cell-cell communication and signaling. The crucial role of ADAM proteases (e.g. ADAM10 and 17) for mammalian development became evident from respective knockout mice, that displayed pre- or perinatal lethality with severe defects in many organs and tissues. Although many substrates for these two ADAM proteases were identified over the last decade, the regulation of their surface appearance, their enzymatic activity and their substrate specificity are still not well understood. We therefore analyzed the constitutive and inducible surface expression of ADAM10 and ADAM17 on a variety of human T cell and tumor cell lines. We demonstrate that ADAM10 is constitutively present at comparably high levels on the majority of the tested cell types. Stimulation with phorbol ester and calcium ionophore does not significantly alter the amount of surface ADAM10, except for a slight down-regulation from T cell blasts. Using FasL shedding as a readout for ADAM10 activity, we show that PKC activation and calcium mobilization are both prerequisite for activation of ADAM10 resulting in a production of soluble FasL. In contrast to ADAM10, the close relative ADAM17 is detected at only low levels on unstimulated cells. ADAM17 surface expression on T cell blasts is rapidly induced by stimulation. Since this inducible mobilization of ADAM17 is sensitive to inhibitors of actin filament formation, we propose that ADAM17 but not ADAM10 is prestored in a subcellular compartment that is transported to the cell surface in an activation- and actin-dependent manner.

## Introduction

In humans, the family of A Disintegrin And Metalloproteases (ADAMs) comprises 21 structurally related transmembrane or secreted proteins, 13 of which are proteolytically active [1]. ADAMs act as ectodomain sheddases for a variety of growth factors or cytokines and the respective receptors and for numerous adhesion molecules. Over the last decade, many different substrates have been identified for individual ADAM proteases and the list is still growing [1]. The prototypic ADAM activity is exerted by ADAM17 (also called ‘tumor necrosis factor α-converting enzyme’, TACE), which is the protease that activates TNF-α by cleaving its pro-form [2–4]. However, meanwhile more than 70 putative substrates for ADAM17 have been identified that include a full array of growth factors and growth factor receptors, cytokines and cytokine receptors, adhesion proteins and respective ligands, or other signaling molecules and their ligands [5]. Interestingly, ADAM proteases display selectivity but also some overlap regarding their substrates. Thus, tissue-, cell type- or activation-dependent expression of ADAMs might provide additional levels of regulation. ADAM17 has been detected in adult organisms in a large variety of tissues including heart, muscle, placenta, ovaries, testes, prostate, pancreas, kidney, small intestine and thymus. In fetal tissues, ADAM17 is prominent in brain, lung, kidney and the liver. Also for ADAM10 more than 25 substrates have been identified over the past years [1,6]. It became apparent that ADAM10 is a key regulator of the Notch and Eph/ephrin pathways and thus is strictly required for embryonic and organismic development [7–12]. ADAM10 is also broadly expressed and is present in fetal brain, liver, heart, kidney and lung, and in lymphoid tissues including bone marrow, thymus, lymph nodes and peripheral blood leukocytes.

For immune cells, we and others have previously shown that ADAM10 is the prominent sheddase for FasL, a TNF-related death factor that plays a pivotal role in T cell death and cytotoxic effector function [13,14]. Shedding of FasL results in the release of a soluble cytokine (sFasL), that supposedly counteracts the apoptosis-inducing capacity of the membrane-anchored (mFasL) death factor [15–17]. For several substrates (including Notch, FasL and TNF), ectodomain shedding by ADAMs leaves N- or C-terminal fragments (NTFs or CTFs) in the plasma membrane that are further processed by secretases or related peptidases in a process termed regulated intramembrane proteolysis (RIP) [18–20]. It is believed that intramembrane proteolysis results in the release of signaling-capable intracellular domains (ICDs) from the N- or C-terminal remnants. For Notch, it has been clearly shown that the proteolytically generated ICDs translocate to the nucleus to regulate gene transcription [11,21,22]. Similar processes have been proposed for the ICDs of TNF and FasL which are processed by the type-2-protein-specific protease SPPL2a [14,23]. Thus, shedding events are not only associated with paracrine effects but also feed back into the protease-containing cell.

Since ADAM family proteases are essential for development and homeostasis, alterations in their activities can be linked to pathology as shown for neurodegenerative disorders, inflammation and cancer. However, so far the regulation of substrate specificity and the mechanisms for activation of most ADAM proteases are not fully understood. ADAM10 seems to be widely associated with the plasma membrane and is transported in Golgi-derived vesicles. ADAM17 was found to be located intracellularly in perinuclear regions and, without stimulation, is much less present on the cell surface. Here, mainly the mature form has been detected which according to some reports seems to associate with cholesterol-rich lipid raft microdomains [24], whereas in other cases ADAM17-mediated shedding is not raft-associated [25].

We investigated the constitutive and inducible surface expression of ADAM10 and ADAM17 on selected tumor cells of different origin and on resting and activated T lymphocytes in a time-frame of three hours without or with stimulation by phorbol ester and calcium ionophore. We observed that ADAM10 is constitutively present on most cell types, even though substantial differences in expression levels were determined by flow cytometry. Surprisingly, for none of the tumor cell lines tested, stimulation altered ADAM10 surface levels. Only for activated T cells blasts and primary T cells, we detected a reproducible decrease in surface abundance over time. However, this mild decrease in ADAM10 levels was not associated with a reduced production of soluble sFasL within the observation period. Nevertheless, an effective down-regulation of ADAM10 using siRNA prevented the production of sFasL and also seemed to decrease the generation of N-terminal fragments to some extent.

In contrast, constitutive ADAM17 expression was very low in general and hardly detectable on transformed or untransformed T cells. Upon phorbol ester and ionophore stimulation, we observed a mild increase in ADAM17 surface expression for some but not all tumor cell lines and for primary and activated T cells. As demonstrated for T cell blasts, this inducible surface expression could be reduced by pharmacological inhibitors of actin filament formation but not by inhibitors of protein synthesis, intracellular protein maturation or ER-to-Golgi transport suggesting a rapid actin-dependent mobilization of ADAM17 from an intracellular storage compartment.

## Materials and Methods

### Cells

Mononuclear cells from peripheral blood (PBMC) were isolated from leukocyte enriched „*Buffy Coat*“ preparations obtained from healthy blood donors at the Institute for Transfusion Medicine, University Hospital Schleswig Holstein Campus Kiel or Campus Lübeck upon approval by the Ethics Committee of the Medical Faculty of the University of Kiel (file reference: D400-10). Primary T cells were enriched from PBMC by rosette formation with neuraminidase-treated sheep red blood cells (SRBC) and density gradient centrifugation. Rosette-forming T cells were collected in the pellet. SRBC were lysed by adding 0.5-1 ml ammonium chloride and incubation for 3-5 minutes. T cells were resuspended and washed thrice in culture medium. PHA blasts were generated by polyclonal stimulation of freshly isolated PBMC with the T cell mitogen PHA (phytohemagglutinin, 0,5 µg/ml, Murex Biotech, Dartford, UK). After three to four days, dead cells were removed by density gradient centrifugation and T cell blasts were transferred to culture medium with rIL-2 (10 U/ml, Chiron, Marburg, Germany) for further expansion. The CD4^+^ T cell clone 12603 has been described elsewhere and was propagated in the presence of feeder cells and rIL-2 (10 U/ml) [26]. The Jurkat-derived cell line JFL39.1 was generated from the apoptosis resistant Jurkat variant J16-Rapo by stable transfection with hFasL [26]. The human pancreatic adenocarcinoma line Panc89 [27] was provided by Prof. Dr. H. Kalthoff (Molecular Oncology, University Hospital Schleswig Holstein Campus Kiel). In addition, we used the following human cell lines: Jurkat (JE6-1, T cell leukemia, ATCC TIB 152), HeLa (cervical carcinoma, DMSZ ACC 075), HT1080 (fibrosarcoma, DMSZ ACC 315), U266B1 (multiple myeloma, DMSZ ACC 009) and L428 (Hodgkin’s lymphoma, DMSZ ACC 197). Cells were kept at 37 °C in humidified atmosphere with 5% (v/v) CO_2_. Suspension cells (PHA blasts, CD4^+^ T cells, JE6-1, J16‑Rapo, JFL39.1, L428, U266B1) and Panc89 were cultivated in RPMI‑1640 with 25 mM HEPES (Invitrogen, Karlsruhe, Germany), 1% (v/v) L-glutamine (Biochrom, Berlin, Germany), 1% (v/v) penicillin/streptomycin (Biochrom) and 10% (v/v) heat-inactivated FCS (Invitrogen). HeLa and HT1080 cells were kept in DMEM with respective supplements. For passaging, these cells were treated with Trypsin/EDTA solution (Biochrom), centrifuged, washed, diluted and further expanded in fresh medium.

### Antibodies

For the detection of ADAM10 and ADAM17 by flow cytometry, we used the following monoclonal antibodies (mAb): α-ADAM10 (AD214Y, SC-73684) was from Santa Cruz Biotechnology, Santa Cruz, CA, USA. The anti-ADAM17 mAb (clone A300E) was generated within the antibody facility (Z3 unit) of the CRC877 [28]. It recognizes a membrane proximal cysteine-rich extension of the ADAM17 protein and was extensively tested in comparison to commercially available antibodies for the use in flow cytometry. PE-labeled anti-mouse secondary antibodies were from Invitrogen and suited isotype controls from Abcam (Cambridge, UK), BD Biosciences (Heidelberg, Germany) or Immunotools (Friesoythe, Germany). For detection of mFasL, we used PE-labeled mAb Alf-2.1 from Invitrogen.

### Chemicals and Inhibitors

To stimulate cells in culture, we used phorbol ester (12-O-tetradecanoylphorbol-13-acetate (TPA) from Sigma-Aldrich, Deisenhofen, Germany) at a final concentration of 10 ng/ml and calcium ionophore (Ionomycin, Calbiochem, Darmstadt, Germany) at 500 ng/ml. Dimethylsulfoxide (DMSO, Merck-Millipore, Darmstadt, Germany) was used as solvent control where indicated. Actin filament formation was inhibited with latrunculin A (2.2 µg/ml, Sigma-Aldrich), or cytochalasin D (5.1 µg/ml, Calbiochem). To block intracellular transport from the ER to the Golgi compartment, we used monensin (2.1 µg/ml, Calbiochem) or brefeldin A (1 µg/ml, Sigma-Aldrich) and to inhibit overall protein synthesis, we used cycloheximide (2 µg/ml, Sigma-Aldrich). In some experiments, we also used the MEK inhibitor U0216 (4.3 µg/ml, Promega, Madison, WI, USA) to block activation of MAPK (ERK1/2). Most inhibitor experiments were performed with PHA blasts that were treated for 30 minutes with individual reagents at the indicated concentrations before stimulation with TPA/Ionomycin.

### Flow cytometric analyses

For the analysis of ADAM proteases, cells growing in suspension were adjusted to 2x10^6^ cells per ml and 100 µl were placed into individual wells of 96 well flat bottom tissue culture plates. Stimulation was induced by successively adding 100 µl of culture medium with TPA and Ionomycin to individual wells. At the end of the incubation period, all cells were transferred to V bottom tissue plates, washed in FACS wash buffer with bovine serum albumin (BSA, Carl Roth, Karlsruhe, Germany) and stained for ADAM10 or ADAM17. Stained cells were washed twice and fixed in 1% PFA (Merck-Millipore) in PBS. Adherent cells were placed in 1 ml of culture medium in 12 well plates one day prior to the stimulation to reach about 70-80% confluency. Respective cell numbers were determined empirically for tumor cells (1x10^5^ per ml for HeLa, 2x10^5^ for HT1080 and 4x10^5^ for Panc89). Stimulation was induced by adding one ml of TPA/Ionomycin solution. At the end of the stimulation period, supernatants were aspirated and the cells were washed once with PBS and then detached with 200 µl accutase (PAA Laboratories, Cölbe, Germany) for 10-30 minutes. The cells were then transferred to microtiter plates for staining as described. For all antibodies, respective isotype-matched controls were included. Of note, during the study we realized that isotype-controls obtained from different companies yielded different background staining which was compensated during setup of the FACSCalibur flow cytometer using CellQuestPro Software (BD Biosciences). Thus, geometric mean values in some experiments may differ based on the isotype control substraction. FasL surface expression on PHA blasts was analyzed as described before using anti-FasL mAbs from Invitrogen.

### Knockdown of ADAM proteases by RNA interference

ADAM10 and/or ADAM17 were knocked down by RNA interference with a set of three validated stealth siRNAs (Invitrogen) using AMAXA^®^ Nucleofector^®^ Technology (Lonza Cologne AG, Cologne, Germany). For hADAM10, ADAM10HSS100167, ADAM10HSS173546 and ADAM10HSS100165 were used and for hADAM17, ADAM17HSS186181, ADAM17HSS110434, and ADAM17HSS110435. For transfection, 5x10^6^ PHA-activated T cell blasts were resuspended in 100 µl human T Cell Nucleofector^®^ solution (Lonza), transferred with 30 nM siRNA into the provided cuvettes and transfected with program X-001 on a Nucleofector^®^ II. After transfection, 500 µl prewarmed culture media were added and the complete sample was transferred to 1.4 ml of prewarmed media in a 12 well culture plate.

### Detection of processed FasL by ELISA

Detection of FasL from supernatants or lysates of PHA blasts was performed using a Human FasL DuoSet^®^ ELISA from R&D Systems following the manufacturer’s protocol. Densitometric analysis was performed on an Infinite^®^ M200 Microplate Reader (Tecan U.S., Durham, NC, USA) at 450 nm (reference 540 nm).

### Western blotting

For Western blotting, cells were harvested at the indicated time points, washed once with ice-cold PBS and resuspended in 1% (v/v) NP-40 lysis buffer supplemented with sodium orthovanadate, sodium fluoride, sodium pyrophosphate, AEBSF, aprotinin, leupeptin and pepstatin A. Following incubation on ice for at least 20 min, cell debris was removed by centrifugation. Equal amounts of protein were then loaded and separated by SDS-PAGE. Upon protein transfer to nitrocellulose membranes (GE Healthcare, Munich, Germany), these were blocked with BSA or dry milk powder. Incubation with primary antibodies was performed for 1 h at room temperature or overnight at 4 °C. Blots were washed three times before adding the horseradish peroxidase-conjugated secondary antibodies. After three additional washes, blots were developed using ECL reagents (GE Healthcare). To detect FasL, we used an anti-FasL mAb (FasLcyto2) generated in our laboratory against the intracellular part of FasL (unpublished, Jing Qian, Dissertation CAU Kiel, 2004) or FAS-L (N-20) polyclonal antibody from Santa Cruz Biotechnology, Santa Cruz, CA, USA. For detection of ADAM10, we used a polyclonal antiserum (from rabbit) which was kindly provided by Dr. Paul Saftig (Biochemical Institute, CAU Kiel) or the anti-ADAM10 mAb 11G2 (Diaclone, Besançon, France). Total ERK1/2 and phosphoERK1/2 were detected with antibodies from Cell Signaling Technology, (Beverly, MA, USA) and GAPDH with a polyclonal antibody from Trevigen Inc. (Gaithersburg, MD, USA) or a mAb (clone 6C5) from Abcam. ADAM17 was detected using the anti-ADAM17 mAb A318, also produced within the antibody core facility of the CRC877.

## Results

### ADAM10 surface abundance and activity

**Figure 1:**
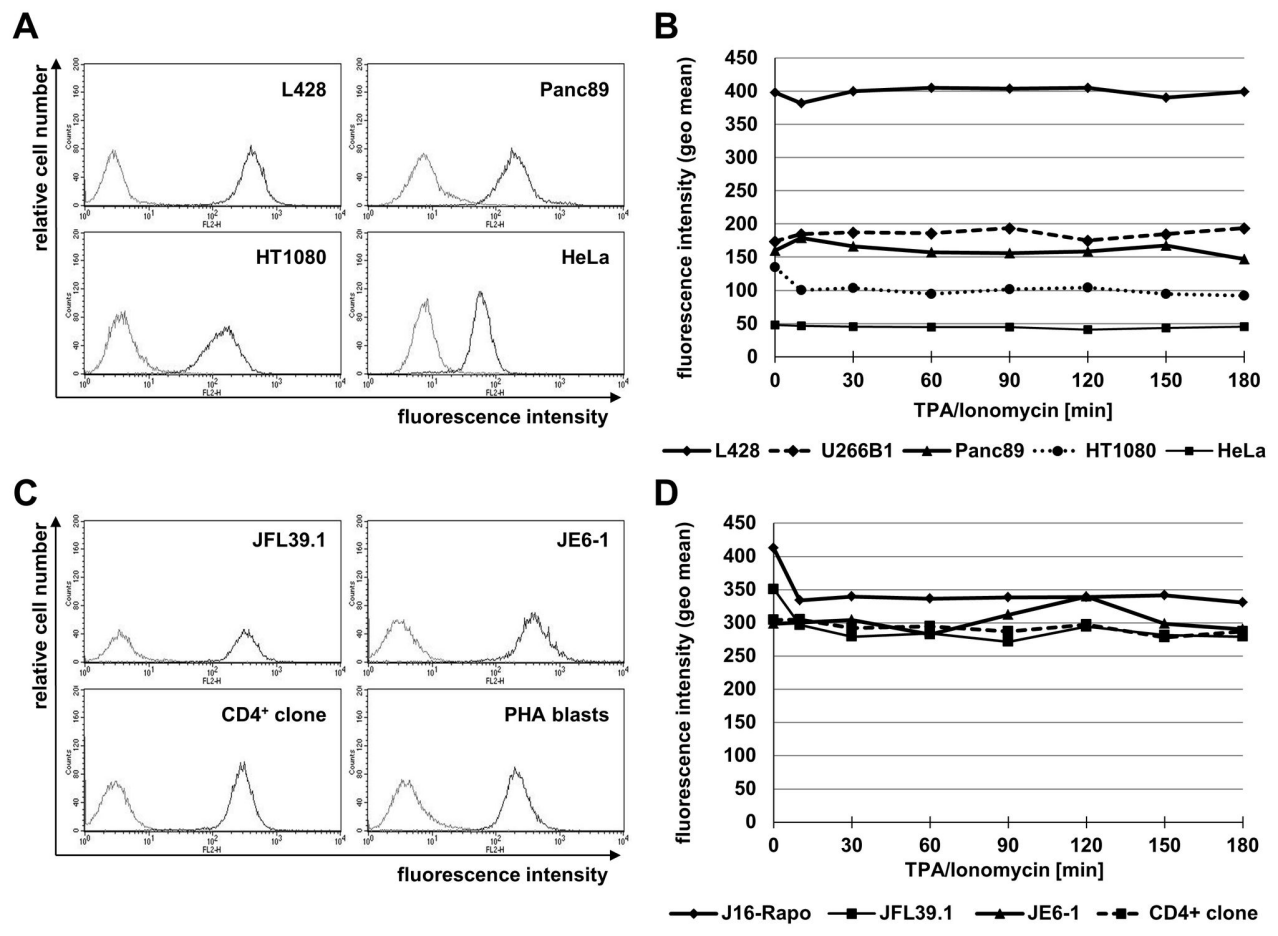
Surface expression and modulation of ADAM10. A. The constitutive surface expression of ADAM10 was monitored on Hodgkin’s Lymphoma (L428), pancreatic ductal adenocarcinoma (Panc89), fibrosarcoma (HT1080) and cervical carcinoma (HeLa) cell lines. Unstimulated cells were stained with an isotype control or anti-ADAM10 mAb (clone AD214Y) and PE-conjugated anti-mouse second step antibodies. Histograms display fluorescence intensities against relative cell numbers. B. Activation-dependent alterations in ADAM10 surface abundance were monitored on tumor cells (also including U266B1 myeloma cells) at the indicated time points after stimulation with TPA and Ionomycin. For each time point, cells were stained with anti-ADAM10 mAb and PE-conjugated anti-mouse second step antibodies. Individual data points represent geometric mean values calculated from individual histograms using CellQuestPro. C. The indicated leukemic and untransformed T cell populations were analyzed for constitutive expression of ADAM10 as described for panel A. D. Activation-induced alterations of ADAM10 on Jurkat variants or cloned T cells were analyzed upon stimulation with TPA and Ionomycin as described for B.

In order to visualize the constitutive surface expression of ADAM10, we analyzed different tumor cell lines and T cell populations by flow cytometry using a mouse mAb raised against the extracellular part of human ADAM10 (clone AD214Y). As shown in Figure 1, all tested cells displayed a relatively high constitutive expression of ADAM10. Using matched settings for the analyses, the Hodgkin’s lymphoma cells (L428) showed the highest relative abundance (calculated geometric mean value 401.62) whereas on other tumor cells, ADAM10 was somewhat less abundant (Panc89 – geo mean 167.51; HT1080 – geo mean 138.14) with the lowest presence on HeLa cells (geo mean 55.18) (Figure 1A). In order to address stimulation-dependent changes in ADAM10 surface expression, we added TPA at 10 ng/ml to activate protein kinase C (PKC) and Ionomycin at 500 ng/ml to induce calcium flux. Whereas it has been reported that ADAM10 activity is primarily modulated by calcium ionophores and less dependent on PKC activation [29,30], the stimulation-dependent modulation of its surface expression has not been systematically addressed. We thus analyzed the surface expression of ADAM10 for up to three hours in the presence or absence of TPA and Ionomycin. As displayed in Figure 1B, the overall surface levels remained unaltered on all tested tumor cells during the observation period, although in some experiments, we observed a slight decrease in the geometric mean values for some cell lines at the onset of the experiment (i.e. after 10 min).

**Figure 2:**
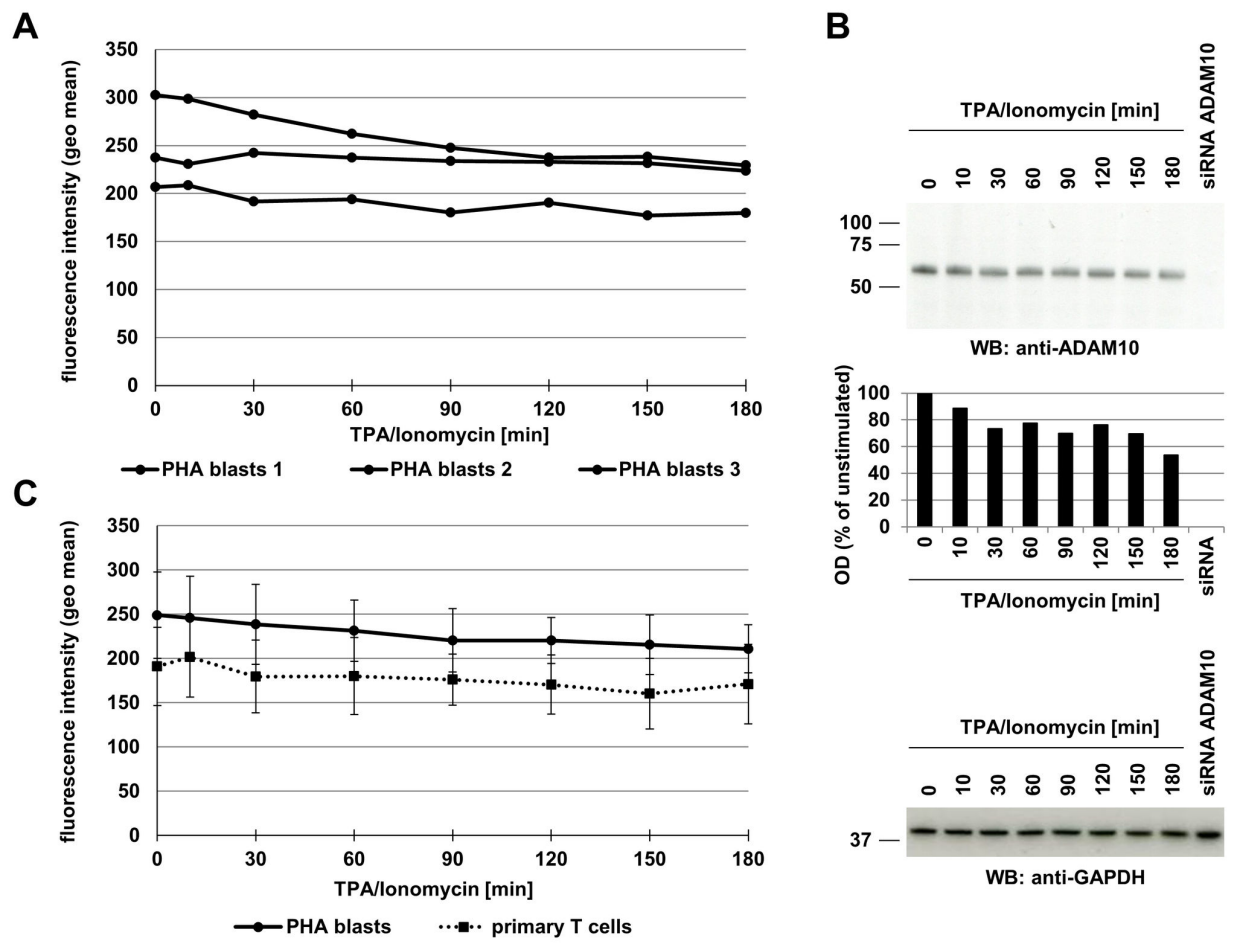
Stimulation-dependent alterations of ADAM10 in untransformed T cell populations. A. PHA-activated T cell blasts from three different donors were stained for ADAM10 at the indicated time points after stimulation with TPA and Ionomycin. Individual data points represent geometric mean values calculated from individual FACS histograms using CellQuestPro. B. PHA-activated T cell blasts were lysed at the indicated time points after stimulation with TPA and Ionomycin and whole cell lysates were separated by SDS-PAGE. ADAM10 was detected with mAb 11G2. A whole cell lysate from ADAM10 siRNA-treated PHA blasts was used as a specificity control. The middle panel shows the densitometric evaluation of the ADAM10 Western blot. Equal loading was tested by re-probing the plot for GAPDH using mAb 6C5 (lower panel). C. The activation-dependent reduction of ADAM10 expression was addressed in freshly isolated T cells or PHA-activated T cell blasts from three individual donors. ADAM10 expression was analyzed by flow cytometry upon stimulation with TPA and Ionomycin. Plotted data represent geometric mean values for individual time points calculated as mean (n=3) including standard deviations.

**Figure 3:**
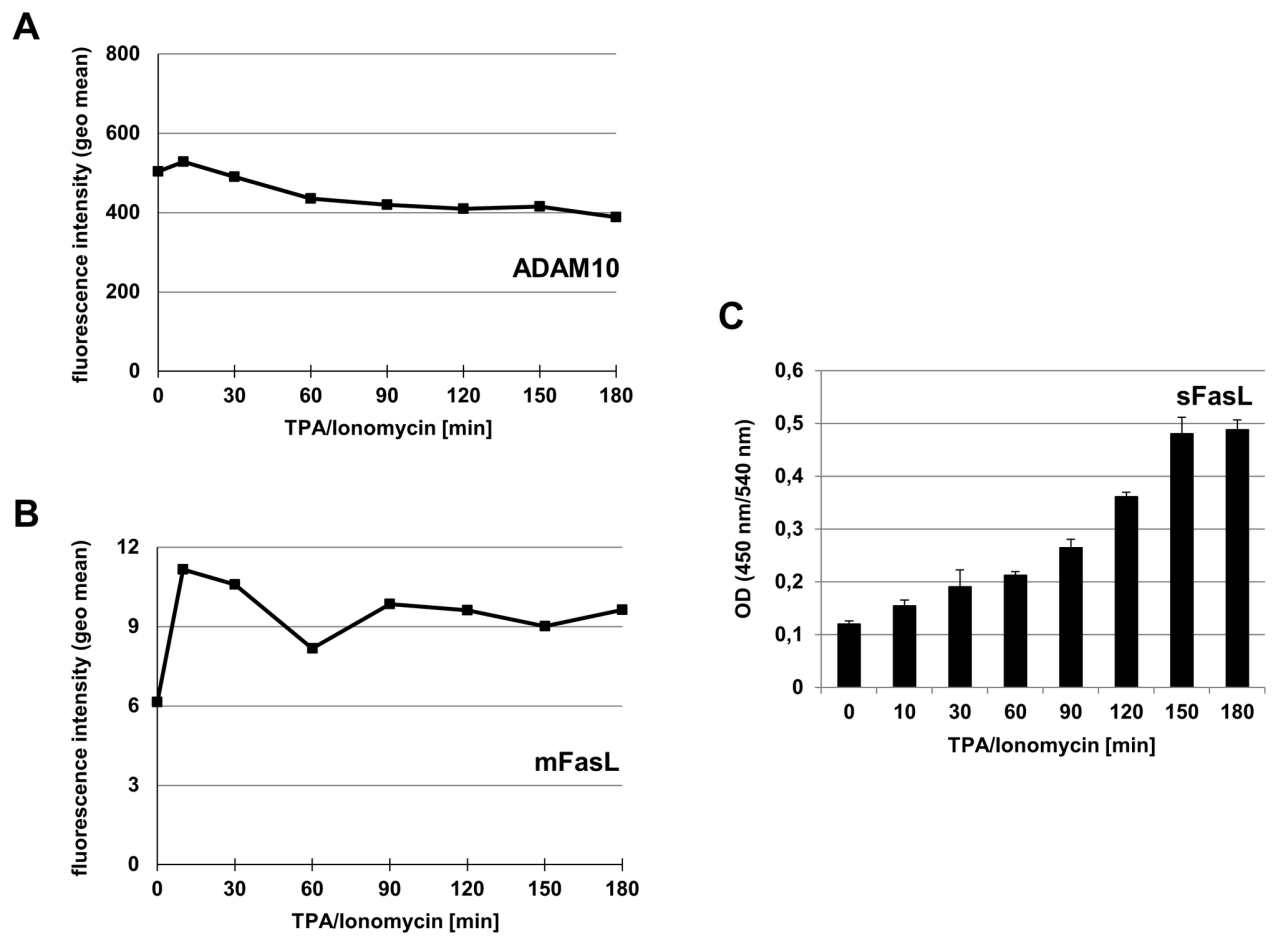
Time-dependent alterations of ADAM10 and FasL in T cell blasts. A. PHA-activated T cell blasts were stained for ADAM10 at the indicated time points after stimulation with TPA and Ionomycin and analyzed by flow cytometry. B. In parallel, FasL surface appearance was monitored using a directly labeled anti-FasL mAb. Individual data points in A and B represent geometric mean values. C. The production (=shedding) of soluble FasL (sFasL) was analyzed in culture supernatants by ELISA as described. Data represent mean values and standard deviations of triplicate cultures.

Also the tested T cell lines and untransformed T cell populations, i.e. Jurkat variants, and cloned CD4^+^ T cells, freshly isolated T cells and PHA blasts, respectively, displayed similar high levels of ADAM10 (Figure 1C, Figure 2). When testing the different T cell populations in the same manner for alterations due to TPA/Ionomycin stimulation, we again did not detect major differences except for a moderate and continuous reduction of ADAM10 on T cell blasts and freshly isolated T cells over time (Figure 1D, Figure 2). This mild decrease of surface ADAM10 was reproducibly detected in PHA blasts and freshly isolated T lymphocytes (Figure 2A,C). Moreover, the reduced level of surface ADAM10 coincided with reduced levels of ADAM10 (but not GAPDH) protein detected in whole cell lysates of stimulated PHA blasts by Western blotting (Figure 2B). We next wanted to address whether this reduction in surface expression would correlate to a reduced shedding of a known ADAM10 substrate. Since we and others had identified FasL as a substrate for ADAM10 in T cells [13,14], we analyzed the presence of surface (membrane) mFasL and soluble sFasL under these conditions (Figure 3). Again, we noted the mild decrease in surface ADAM10 (Figure 3A). In parallel, TPA/Ionomycin stimulation resulted in a biphasic profile of mFasL surface appearance in T cell blasts, as we had reported for purified CD4^+^ or CD8^+^ T cell populations before [15,17,31] (Figure 3B). Interestingly, although the mild decrease in ADAM10 was reproducibly detected in all experiments, this did not coincide with a reduced production of sFasL as determined by ELISA (Figure 3C).

**Figure 4:**
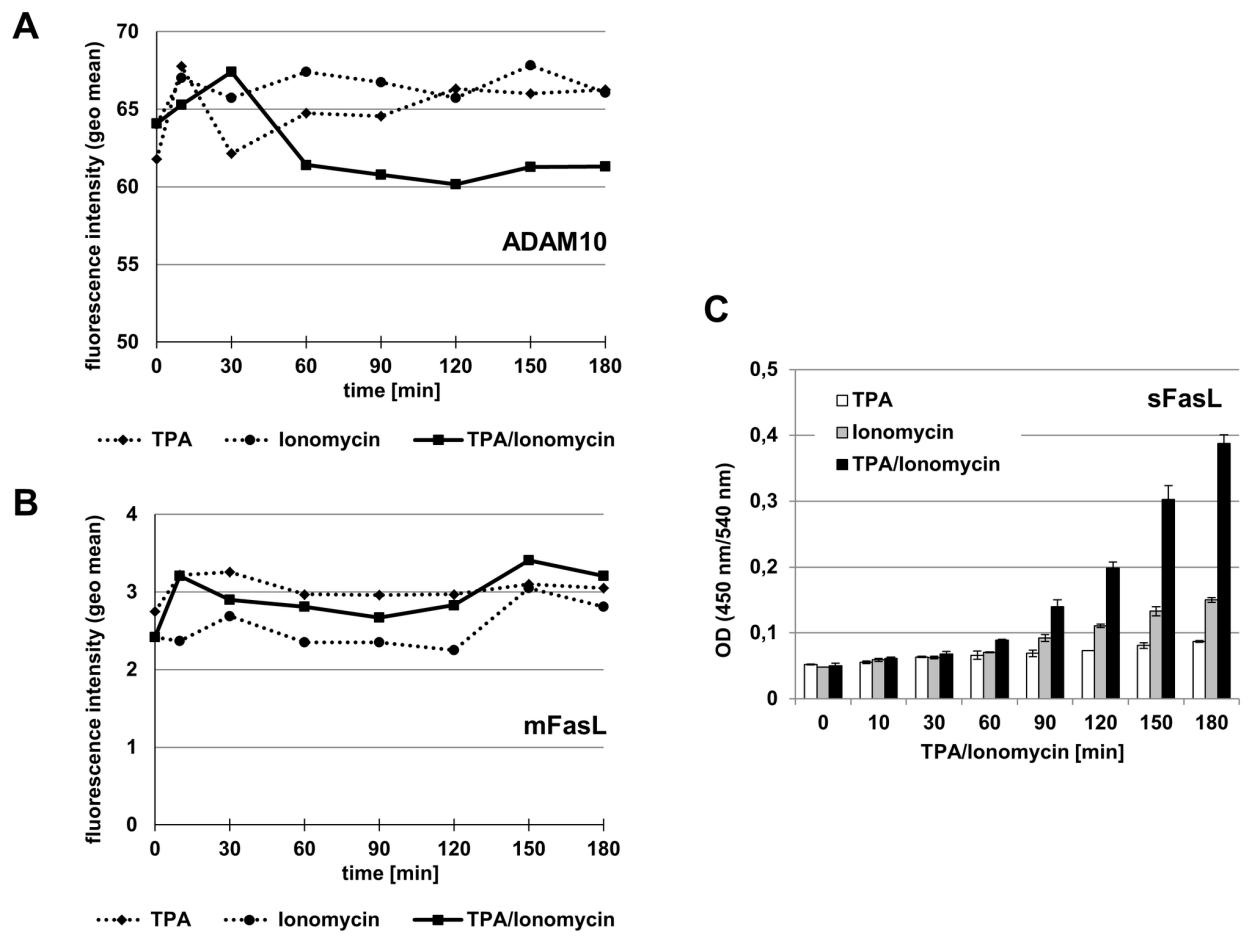
Phorbol ester and calcium ionophore are required for ADAM10 down-modulation and the production of sFasL. A. PHA-activated T cell blasts were stained for ADAM10 at the indicated time points after stimulation with TPA and Ionomycin or with TPA or Ionomycin alone. B. FasL surface appearance was monitored in parallel. Individual data points in A and B represent geometric mean values calculated from individual histograms using CellQuestPro. C. At the indicated time points, culture supernatants were tested for the presence of shed soluble FasL (sFasL) by ELISA. Data represent mean values and standard deviations of triplicate cultures.

Next, we were interested whether PKC activation or calcium mobilization would be required for ADAM10 activity, again using sFasL production as primary readout. In the same experimental setting, we thus incubated the T cell blasts with TPA alone, with Ionomycin alone or using a combination of the two compounds. As depicted in Figure 4A, both factors were needed to reduce the surface level of ADAM10. For mFasL, the effects were moderate with a tendency of slightly lower mFasL expression in the presence of Ionomycin (Figure 4B). Notably, whereas TPA had almost no impact on the production of sFasL within the three hours observation period, Ionomycin alone resulted in a mild increase of soluble sFasL after one hour (Figure 4C). Most impressively, however, the combination of PKC activation and calcium mobilization resulted in a drastic enhancement in sFasL production (Figure 4C).

**Figure 5:**
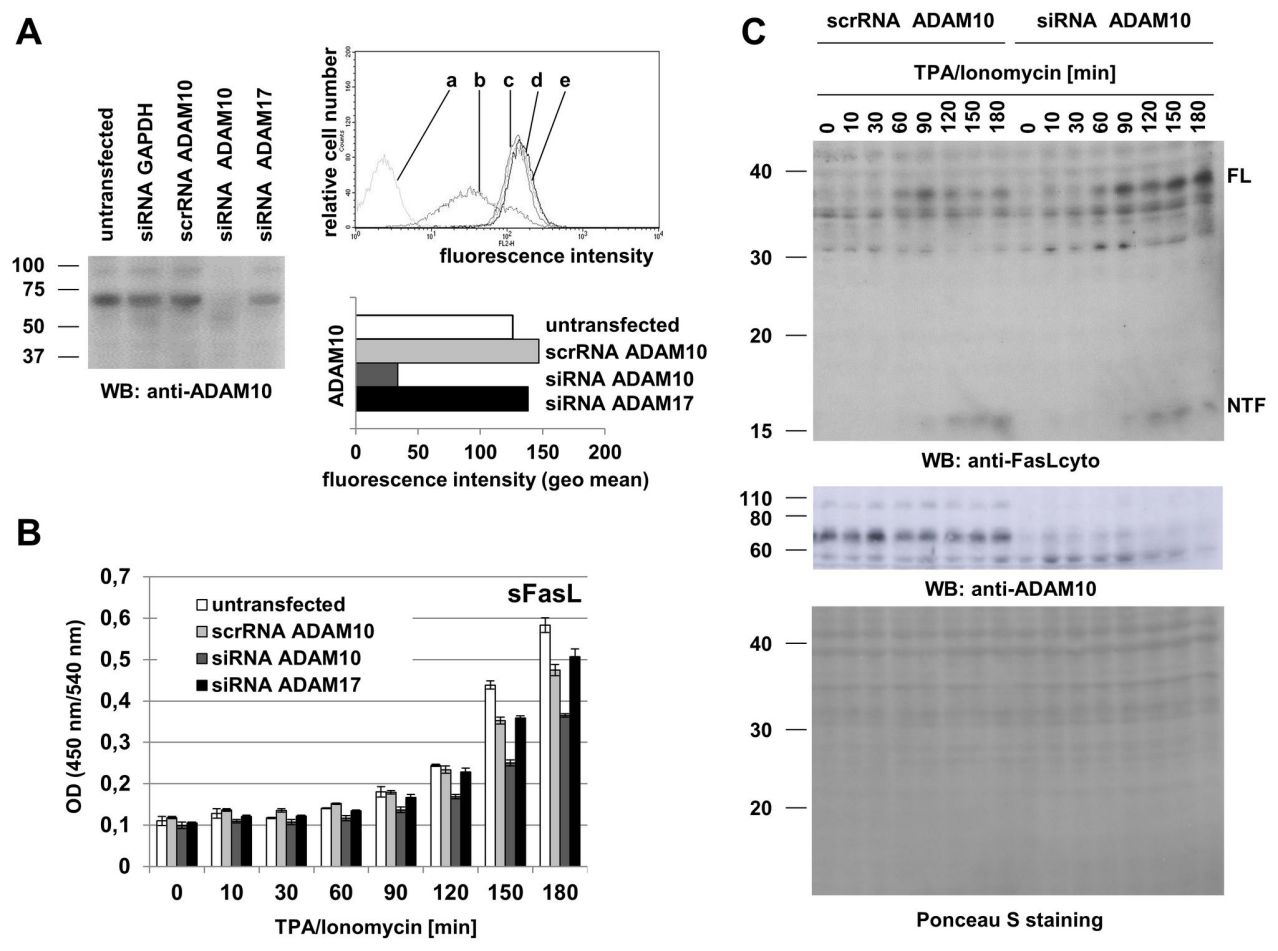
Knockdown of ADAM10 by siRNA blocks the production of sFasL. A. The efficacy of the ADAM10 knockdown was monitored by Western blotting of whole cell lysates from untransfected or siRNA-transfected PHA blasts (left panel). Here, the level of surface ADAM10 was significantly reduced in ADAM10 siRNA-treated cells but not in the respective controls. Moreover, surface ADAM10 was analyzed by flow cytometry using a respective isotype control (light grey line, a) and mAb AD214Y. ADAM10 expression was only reduced in ADAM10 siRNA-treated cells (black dotted line, b), but not in untransfected (dark grey line, c), control siRNA- (black line, d) or ADAM17 siRNA-treated (dashed dark grey line, e) PHA blasts (upper right panel). Data in the bar graph (lower right panel) represent geometric mean values calculated from the individual histograms. B. In addition, culture supernatants collected at the indicated time points were tested for the presence of soluble FasL by ELISA. As expected, sFasL production was most prominently affected in ADAM10 siRNA-treated cells. C. In order to analyze the presence of full length (FL) FasL and its N-terminal fragments (NTF), scrRNA- and ADAM10 siRNA-transfected T cell blasts were lysed at the indicated time points after stimulation with TPA and Ionomycin. FasL was detected using a mAb (FasLcyto2) generated in our laboratory against the intracellular region of the molecule and thus allowing the simultaneous detection of full length FasL (FL) and proteolytically generated N-terminal fragments (NTF) (upper panel). The ADAM10 knockdown was monitored by reprobing the blot with a polyclonal anti-ADAM10 antibody (middle panel). Equal protein loading and transfer was routinely checked by Ponceau S staining (lower panel).

In previous experiments, FasL shedding was assigned to ADAM10 activity on the basis of more or less specific pharmacological inhibitors for ADAM10 and ADAM17 [13]. In order to confirm that ADAM10 is the main sheddase for FasL, we thus wanted to reduce the amount of ADAM10 protein by RNA interference. To this end, we used a set of stealth siRNAs targeting ADAM10 or ADAM17 for comparison. The combination of three ADAM10-specific siRNAs very efficiently reduced the level of ADAM10 detected in whole cell lysates and on the cell surface (Figure 5A). Notably, siRNAs against ADAM17 left surface ADAM10 largely untouched. When we addressed the production of sFasL over time by ELISA, we observed a reduction in the absence of ADAM10 but not when ADAM17 was targeted (Figure 5B). At the same time, we detected an accumulation of full length FasL by Western blotting in lysates from ADAM10 siRNA-treated cells and a mildly reduced formation of FasL N-terminal fragments (Figure 5C).

Taken together, we show that ADAM10 is expressed at relatively high levels on most tested cells. Its surface expression is not significantly altered in the presence of TPA and Ionomycin. In T cell blasts, ADAM10 levels mildly decline with stimulation. In terms of activation and substrate cleavage, ADAM10 activity can be most efficiently induced by combining PKC activation and calcium mobilization. Importantly, the siRNA-induced knockdown of ADAM10 confirmed that ADAM10 is a major sheddase for FasL in activated T cells.

### ADAM17 Surface Expression

**Figure 6:**
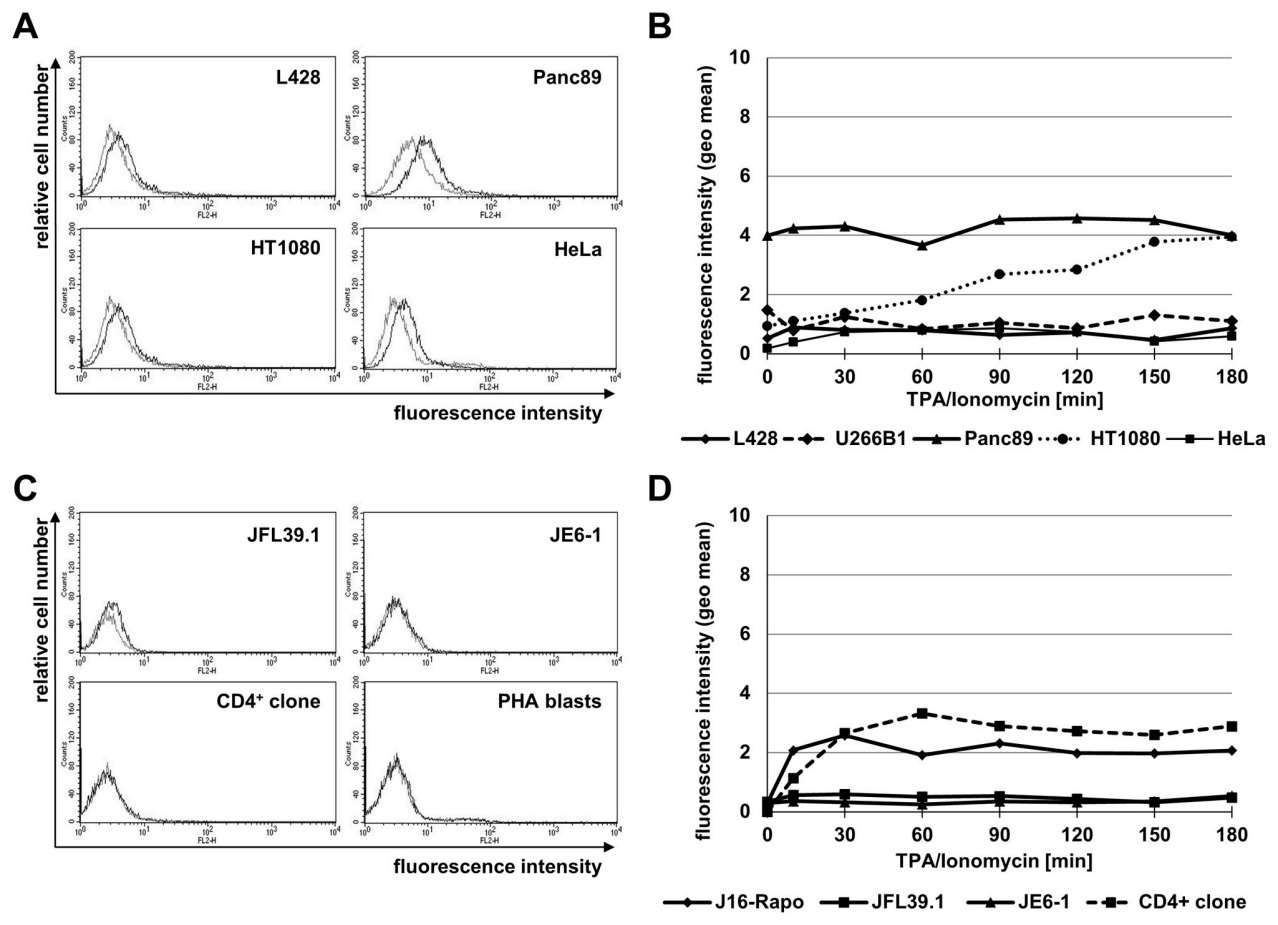
Surface expression and modulation of ADAM17. A. The constitutive surface expression of ADAM17 was monitored on Hodgkin’s Lymphoma (L428), pancreatic ductal adenocarcinoma (Panc89), fibrosarcoma (HT1080) and cervical carcinoma (HeLa) cell lines. Unstimulated cells were stained with an isotype control or anti-ADAM17 mAb (clone A300E) and PE-conjugated anti-mouse second step antibodies. Histograms display fluorescence intensities against relative cell numbers. B. Activation-dependent alterations in ADAM17 surface abundance were monitored on the listed tumor cell lines (including U266B1) at the indicated time points after stimulation with TPA and Ionomycin. For each time point, cells were stained for ADAM17 as described. Individual data points represent geometric mean values calculated from individual histograms using CellQuestPro. C. The indicated leukemic and untransformed T cell populations were analyzed for constitutive expression of ADAM17. D. Activation-induced modulation of ADAM17 was analyzed for a period of three hours upon stimulation with TPA and Ionomycin on transformed Jurkat variants and an untransformed T cells.

**Figure 7:**
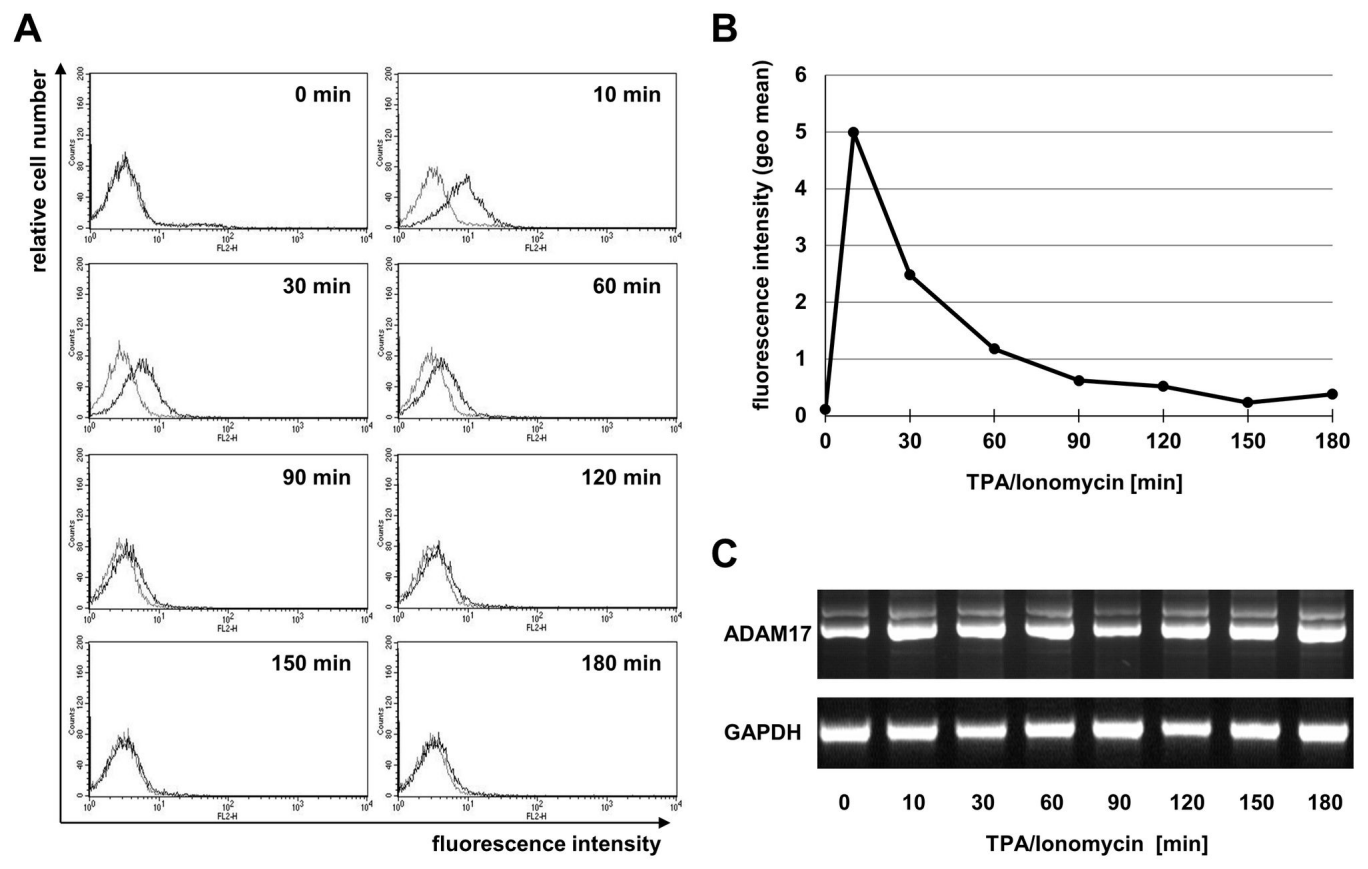
ADAM17 alterations in stimulated PHA blasts. PHA-activated T cell blasts were stained for ADAM17 at the indicated time points after stimulation with TPA and Ionomycin using mAb A300E. A. Histograms for individual data points document the rapid and transient induction of surface ADAM17. B. Visualization of ADAM17 kinetics based on the calculation of geometric mean values from individual histograms shown in A.C. The RT-PCR analyses for ADAM17 and GAPDH as a control performed on samples obtained in parallel at the indicated time points did not reveal any indication for changes at the level of mRNA.

**Figure 8:**
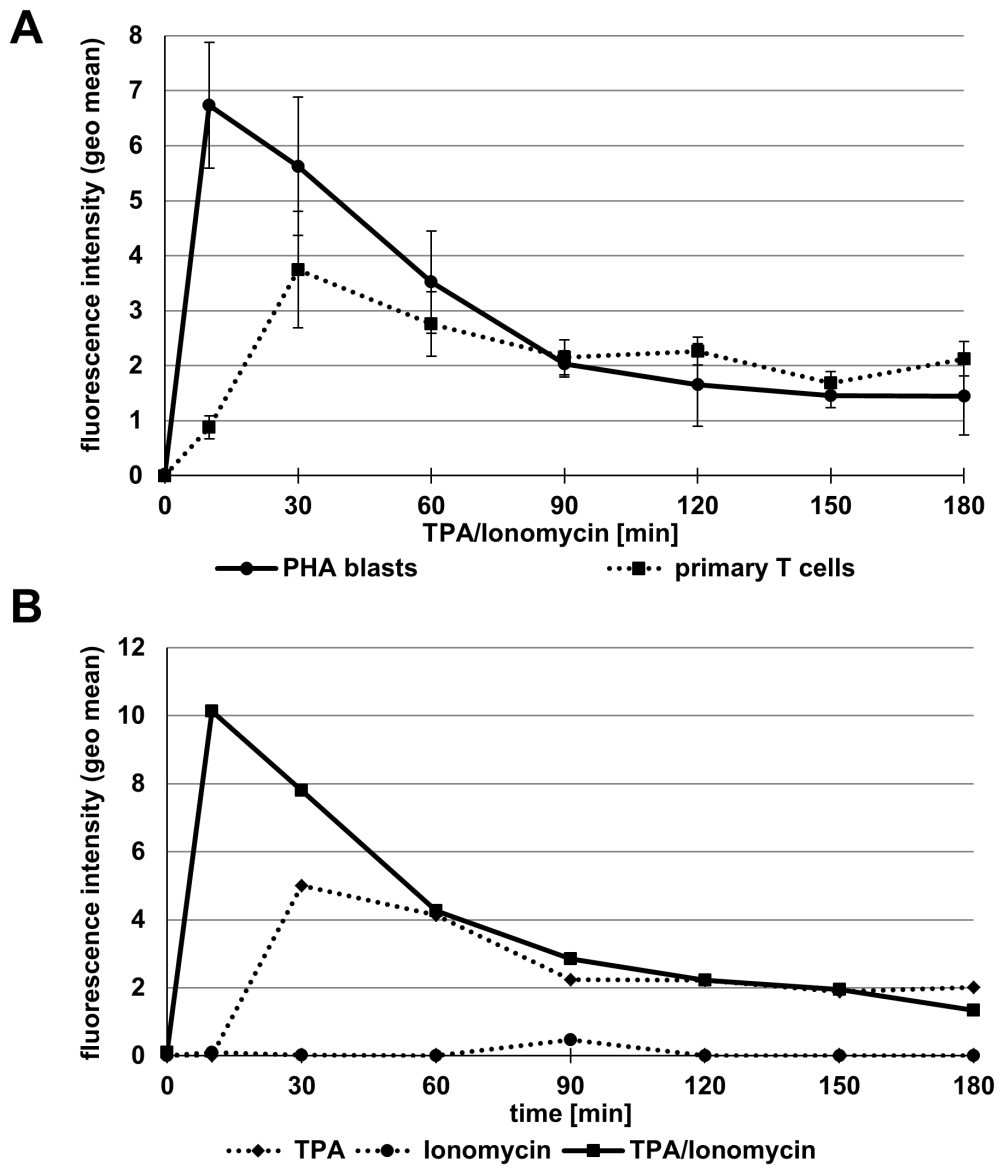
ADAM17 induction in freshly isolated and *in* *vitro* **expanded T cells**. A. PHA-activated T cell blasts and freshly isolated primary T cells from three different individuals were stained for ADAM17 at the indicated time points after stimulation with TPA and Ionomycin. In all cases, we observed an increase of ADAM17 on the cell surfaces. Mean values (n=3) and error bars are given based on geometric mean values calculated from individual FACS histograms. Apparently, in pre-activated T cell blasts ADAM17 induction is more rapid and reaches a higher amplitude as compared to freshly isolated T cells. B. In order to address the role of TPA versus Ionomycin for the induction of ADAM17, PHA blasts were stained for anti-ADAM17 at the indicated time points after stimulation with either TPA or Ionomycin or both stimuli. Data are shown as geometric mean values for one representative experiment.

In parallel, we addressed the expression of ADAM17 on the same set of tumor cells and T cell lines. As shown in Figure 6, in comparison to ADAM10, all tested cells displayed a considerably lower constitutive expression of ADAM17 (Figure 6 A,C). When we addressed stimulation-dependent alterations in ADAM17 surface levels, we noted a significant increase in ADAM17 after incubation with phorbol ester and Ionomycin on individual tumor cell lines (i.e. the fibrosarcoma line HT1080, Figure 6B) and some tested T cell populations (Figure 6D). We therefore followed the stimulation-dependent ADAM17 expression on PHA blasts in more detail and detected an even more prominent surface appearance of ADAM17 (Figure 7A,B). Highest expression was seen after 10 minutes. When plotted as time-dependent alterations of fluorescence intensity, surface appearance of ADAM17 was transient and started to decline rapidly. Importantly, the level of mRNA analyzed by RT-PCR remained constant throughout the incubation period of three hours (Figure 7C). Similar kinetics were reproducibly seen in all independent experiments using PHA blasts and freshly isolated T cells (Figure 8A). As indicated, surface appearance in most cases was slightly faster on activated PHA blasts as compared to freshly isolated T cells. In addition, also the amplitude of surface ADAM17 was higher in T cell blasts. When T cell blasts were stimulated with TPA or Ionomycin alone or in combination, we observed that calcium ionophore did not suffice to induce ADAM17 expression. In contrast, TPA alone induced some increase in ADAM17, which was nevertheless further enhanced in speed and amplitude when both stimuli were used in combination (Figure 8B).

### ADAM17 is mobilized to the cell surface in an actin-dependent manner

**Figure 9:**
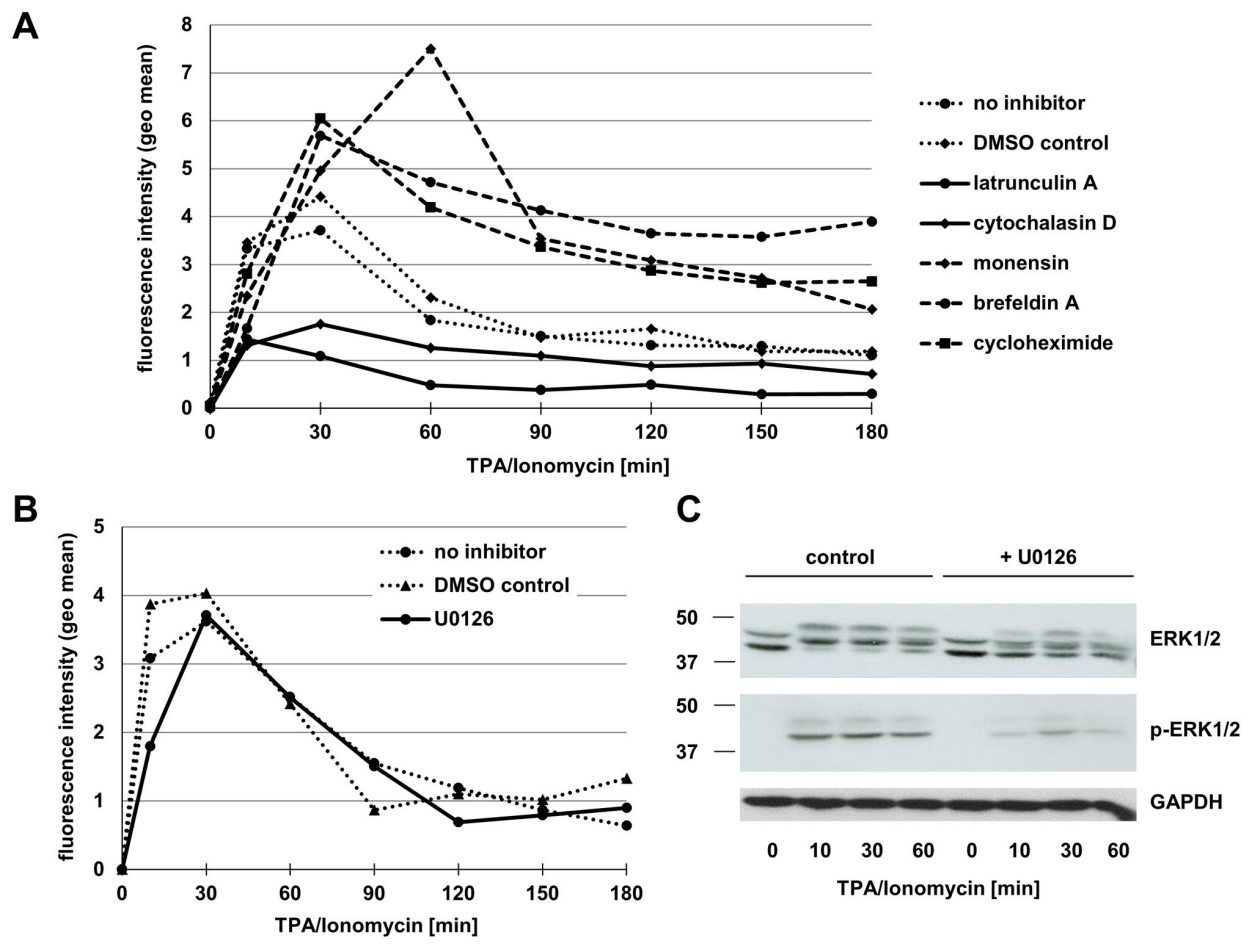
ADAM17 up-regulation can be blocked by inhibitors of actin filament formation. A. PHA blasts were stained for ADAM17 at the indicated time points after stimulation with TPA and Ionomycin with or without pretreatment with the indicated inhibitors for 30 minutes. Only latrunculin A and cytochalasin D markedly reduced the level of inducible ADAM17. B. PHA-activated T cell blasts were stained for ADAM17 at the indicated time points after stimulation with TPA and Ionomycin in the presence or absence of the MEK inhibitor U0126 or DMSO as a solvent control. ERK activation did not influence inducible ADAM17 induction in PHA blasts. C. Western blot for ERK1/2, phospho-ERK1/2 (p-ERK1/2) and GAPDH from T cell blasts. PHA-activated T cell blasts were stimulated for the indicated time periods with TPA and Ionomycin in the presence or absence of U0126 or DMSO as a control. Cells were lysed and proteins separated by SDS-PAGE were analyzed for total ERK protein and ERK phosphorylation. GAPDH staining served as a loading control.

**Figure 10:**
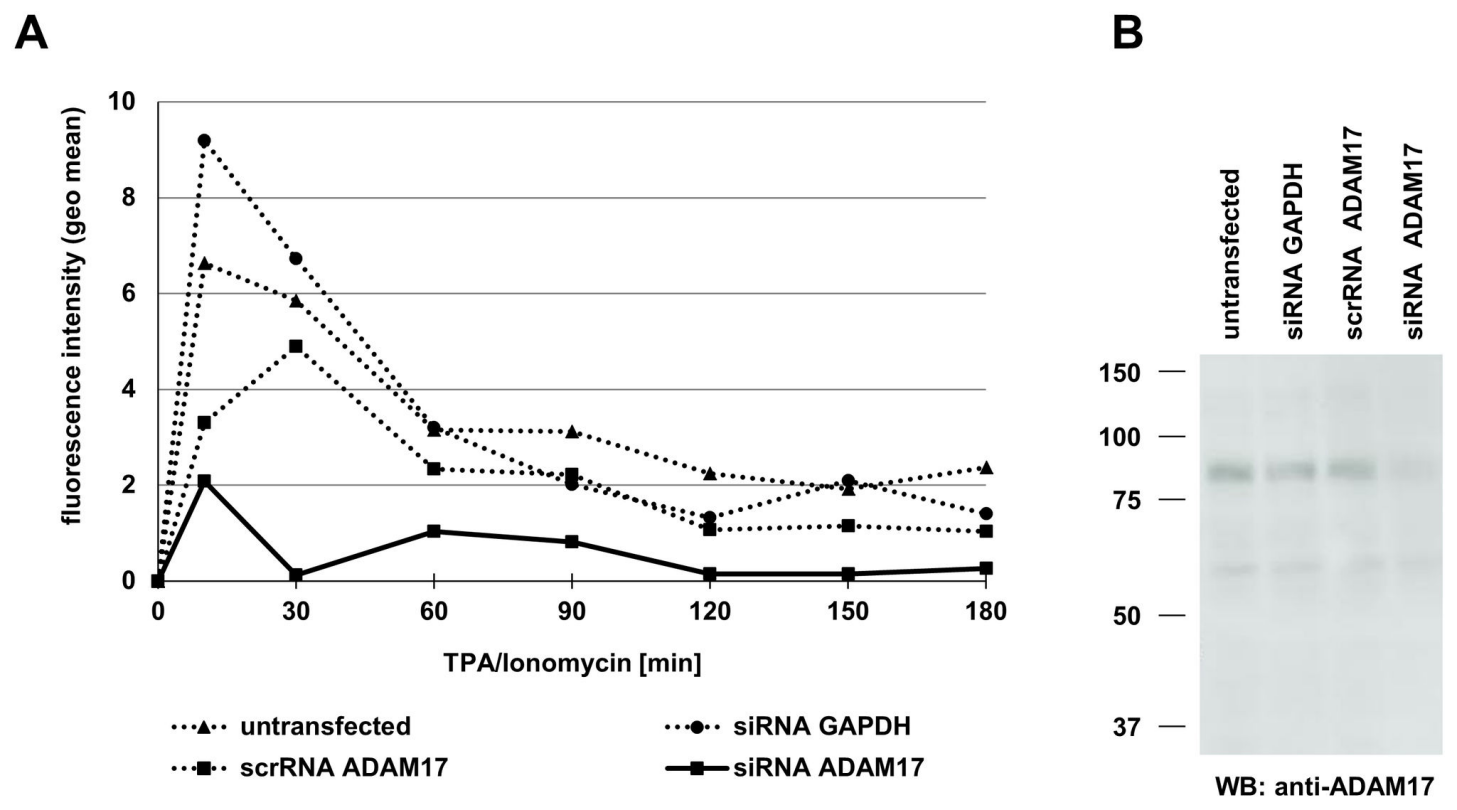
Knockdown of ADAM17 prevents inducible ADAM17 surface transition. A. PHA-activated T cell blasts were transfected or not with the indicated control or specific siRNAs. After 72 hours, the cells were stained for ADAM17 at the indicated time points after stimulation with TPA and Ionomycin. B. ADAM17 siRNA knockdown was verified at the protein level by Western blotting using mAb A318.

A rapid surface appearance of preformed proteins in T cells might be associated with an induced degranulation or the mobilization of storage vesicles [17,26,31,32]. These processes often require a reorganization of cytoskeletal elements such as actin filaments. Therefore, we tested the surface appearance of ADAM17 in the presence or absence of pharmacological inhibitors of actin filament formation such as latrunculin A and cytochalasin D. Indeed, both inhibitors markedly reduced the inducible surface expression of ADAM17 in T cell blasts (solid lines in Figure 9A). In contrast, inhibitors of protein biosynthesis (cycloheximide), ER-to-Golgi transport (brefeldin A) and general protein transport (monensin) did not exert a comparable inhibitory effect but rather increased surface ADAM17 as compared to the controls (dashed versus dotted lines in Figure 9A). Also inhibition of the ERK signaling pathway applying U0126 to inhibit MEK did not affect the inducible up-regulation of ADAM17 on PHA blasts (Figure 9B) or primary T cells (not shown), although Western blotting revealed a clear reduction of ERK phosphorylation in PHA blasts (Figure 9C). Finally, we also tested whether a knockdown of ADAM17 by siRNA would abrogate the inducible surface appearance. As depicted in Figure 10, only the transfection with the ADAM17 specific siRNA resulted in a reduction of inducible surface expression (Figure 10A) associated with a markedly reduced total protein level of mature ADAM17 as detected by Western blotting using mAb 318 (Figure 10B).

Taken together, we demonstrate that ADAM17 is constitutively only weakly associated with the cell surface. However, ADAM17 can be upregulated on some tumor cells and on T lymphocytes by stimulation with TPA and Ionomycin. Surface appearance is transient and can be blocked by inhibitors of actin filament formation but not by MEK inhibitors.

## Discussion

Although ADAM10 and ADAM17 are often regarded as close relatives within the ADAM protease family, our analyses revealed striking differences with respect to their constitutive and inducible surface expression on transformed and non-transformed cell populations. While without stimulation ADAM10 was found at comparably high levels on all cell types analyzed (Figure 1), ADAM17 was almost not detectable on T cells and only present at low levels on some tumor cell types, e.g. pancreatic ductal adenocarcinoma (Panc89) and fibrosarcoma (HT1080) cell lines (Figure 6). Regarding the expression of ADAM10 on tumor cell lines of different origin, we observed the highest levels of ADAM10 on the Hodgkin’s lymphoma cell line L428 and on leukemic Jurkat variants, followed by myeloma and pancreatic carcinoma cells (Figure 1). Relatively little constitutive ADAM10 was seen on fibrosarcoma and cervical carcinoma cells. This might indicate that hematopoietic tumor cells display more constitutive ADAM10 as compared to non-hematopoietic cells.

Using phorbol ester and calcium ionophore as stimuli with documented effects on the regulation of ADAM protease activity and expression [29,30,33–36], we did not observe a major effect on the surface levels of ADAM10 on tumor cells (Figure 1). For untransformed T lymphocytes, we reproducibly observed a mild constant decline over the observation period of three hours (Figure 2). This mild reduction was also seen in Western blotting at the level of total protein but apparently did not coincide with a considerable loss of activity, e.g. production of sFasL (Figures 3-5). This might indicate that ADAM10 itself is regulated to some extent by proteolytic processing, e.g. by other ADAM family proteases such as ADAM9 or ADAM15 [19,37] or by activation-dependent internalization. Looking at FasL production as a readout, we addressed signal requirements for ADAM10-associated substrate processing. We could show that PKC activation or calcium mobilization alone are not sufficient for the processing of FasL. Both stimuli are required to induce significant levels of sFasL (Figure 4). Importantly, siRNA-mediated knockdown of ADAM10 clearly confirmed that ADAM10 is the major sheddase for FasL in activated T cells (Figure 5). The knockdown reduced ADAM10 surface and total protein levels and consequently the production of sFasL. In addition, using a mAb directed against the intracellular part of FasL for Western blotting, we detected an increase of full length FasL and a mild relative reduction in the formation of its N-terminal fragments in the absence of ADAM10 (Figure 5).

Surface levels of ADAM17 were much lower on all tested cells. Employing the anti-ADAM17 mAb A300E for flow cytometry, all transformed and untransformed T cell lines were essentially negative for ADAM17. Significant constitutive surface expression was only detectable on pancreatic adenocarcinoma cells (Panc89) and little expression was detected on fibrosarcoma cells (HT1080) (Figure 6). Upon stimulation, we observed an increase of ADAM17 in HT1080 cells, and no significant change in the Hodgkin’s lymphoma cell line L248, HeLa cells, U266B1 myeloma cells, Panc89 or Jurkat variants. Comparing the calculated geometric mean values, one should stress that also the levels of ADAM17 detected after stimulation remain minute when compared to the level of ADAM10.

ADAM proteases are supposed to be translated as inactive pro-enzymes, containing a pro-domain that works as an intramolecular chaperone, keeping the protease in an inactive conformation. During maturation in late Golgi vesicles, the pro-domains are believed to be cleaved off by furin or pro-protein convertases. The exact mechanisms how ADAM10 and 17 are stored, activated or transported to the plasma membrane, however, are still unsolved. It was reported that in the case of ADAM17, ERK-mediated phosphorylation of Thr735 might promote surface appearance [38]. However, in our experiments reduced ERK phosphorylation did not impair the inducible ADAM17 surface expression. Only recently, it was reported that an inactive member of the rhomboid family of proteases (iRhom2) is needed for ADAM17 translocation to the cell surface [39,40].

Like other ADAM proteases, ADAM10 and 17 are glycosylated and they contain several regulatory regions in their intracellular tails including putative ER retention and proline-rich SH3 domain interaction motifs. Although most sketches display active ADAM proteases at the cell surface, it has been proposed that some proteases might cleave certain substrates in intracellular compartments from the ER to late Golgi-derived vesicles [41–43]. Our findings suggest that at least ADAM17 might be stored in some intracellular storage compartment. Along this line, preliminary results employing a recently described protocol for the enrichment and characterization of distinct lysosomal compartments [44–47], suggest that in fact both ADAM10 and ADAM17 might associate with vesicular structures in T cells (our own unpublished results, manuscript in preparation).

The enzymatic activity of ADAM proteases is inducible by a variety of different stimuli [6,48,49] including activation of G-protein coupled receptors [50,51], activators of protein kinase C (PKC) [52–54], calcium ionophores [35,36], bacterial toxins [55] and apoptotic stimuli [56]. Responsiveness of individual ADAMs to certain stimuli seems to vary considerably. Moreover, also a number of other natural or experimental activation processes have been reported to be associated with increased protease activity and can synergize to increase the shedding of a given substrate. For several ADAMs, a constitutive shedding is discriminated from an inducible shedding and occasionally this can affect the same substrate and its cleavage by two different ADAM proteases. One example would be the cleavage of the adhesion molecule L1, which is constitutively shed by ADAM10 but will be cleaved by ADAM17 after TPA-treatment and activation of PKC [57,58]. This and other observations led to the assumption that ADAM10 might be generally less susceptible to TPA-induced activation than ADAM17.

Although stimulation by phorbol ester and/or calcium ionophore alters shedding activities, it is yet not clear whether this activation relates to changes in the localization of the enzymes and substrates, i.e. recruitment to the plasma membrane or repositioning into lipid raft areas or tetraspanin-associated microdomains, or to conformational changes facilitating proteolysis. Since phorbol ester application (PKC activation) stimulates protein secretion and has also been implicated in the mobilization of ADAM proteases (e.g. ADAM12 from intracellular storage compartments [59,60]), various mechanisms could contribute to the rapidly inducible shedding by ADAM17. Interestingly, also the reported association of ADAM10 and ADAM17 with repositioning and activity promoting tetraspanins seems to differ between the two proteases. Whereas ADAM10 interacts with several members of the conserved TspanC8 subfamily (Tspan5, Tspan10, Tspan14, Tspan15, Tspan17, and Tspan33) and Tspan12 [61–63], ADAM17 was shown to interact primarily with CD9 [64]. Notably, over the past years, this PKC issue has been rather controversially discussed: A number of reports indicated that PKC and/or growth factor stimulation result in an ERK-dependent phosphorylation of the intracellular part of ADAM17 [38,65–67]. It was proposed that this phosphorylation might be prerequisite for the maturation of ADAM17 and for its translocation to the plasma membrane. However, several other studies did not find any significant impact of phorbol ester on the maturation or surface distribution of ADAM17. Dodens & Black even reported an internalization of ADAM17 one hour after TPA treatment [68]. In our own experiments, we observed that TPA activation alone also resulted in an increased ADAM17 expression, which, however was further enhanced and prolonged in the presence of the calcium ionophore (Figure 8B).

Whereas several Jurkat variants did not react to TPA/Ionomycin stimulation with significant changes in the levels of ADAM17, we found that untransformed T cell populations reproducibly showed a rapid and transient increase in surface ADAM17 (Figures 7 and 8). This was best seen in PHA-activated T cell blasts and was not associated with detectable alterations at the level of mRNA (Figure 7). Regarding the amplitude and kinetics of ADAM17 expression, T cell blasts reacted more rapidly than freshly isolated cells and also showed a higher maximal expression (Figure 8). A rapid induction of surface expression is often associated with the activation-induced translocation of vesicular/lysosomal storage compartments to the cell surface. One typical example for T and NK cells would be the recruitment of secretory lysosomes to the immunological synapse when facing a putative target cell [69,70]. Thus, we addressed whether one could block the surface transition of ADAM17 using pharmacological inhibitors for protein synthesis or intracellular protein transport. Whereas cycloheximide, monensin or brefeldin A did not interfere with the induction of ADAM17, cytochalasin D and latrunculin A effectively reduced the mobilization of ADAM17 to the cell surface (Figure 9A). Also, the MEK inhibitor U0126 did not influence ADAM17 transition although it reduced ERK phosphorylation as revealed by Western blotting for phospho-ERK (Figure 9B,C). As shown in Figure 9A, the thirty minutes pre-incubation with cycloheximide, monensin or brefeldin A seemed to enhance ADAM17 surface expression. Although this observation is counterintuitive, similar effects were seen in several independent experiments. Here one might speculate that the pre-incubation with these reagents induces some unspecific membrane alterations that influence subsequent signal transduction events. Notably, if one blocks ADAM17 protein translation by RNA interference, the inducible surface appearance is almost completely abrogated (Figure 10).
